# A Blueprint for the Problem Formulation Phase of EPA-Type Ecological Risk Assessments for 316(b) Determinations

**DOI:** 10.1100/tsw.2002.862

**Published:** 2002-06-25

**Authors:** Webster Van Winkle, William P. Dey, Steve M. Jinks, Mark S. Bevlhimer, Charles C. Coutant

**Affiliations:** Van Winkle Environmental Consulting Co., 5163 N. Backwater Ave., Boise, ID 83703, USA; ASA Analysis & Communication, Inc., 291 County Road 62, New Hampton, NY 10958, USA; Environmental Sciences Division, Oak Ridge National Laboratory, Oak Ridge, TN 37831-6036, USA

**Keywords:** analysis plan, assessment endpoint, conditional mortality rate, cooling-water intake system, ecological risk assessment, entrainment, equivalent loss, exposure and effects, fish population, fractional loss, impingement, individual loss, management objective, measure, prospective analysis, power plants, problem formulation, retrospective analysis, representative species

## Abstract

The difference between management objectives focused on sustainability of fish populations and the indigenous aquatic community, and a management objective focused on minimizing entrainment and impingement losses accounts for much of the ongoing controversy surrounding §316(b). We describe the EPA’s ecological risk assessment framework and recommend that this framework be used to more effectively address differences in management objectives and structure §316(b) determinations. We provide a blueprint for the problem formulation phase of EPA-type ecological risk assessments for cooling-water intake structures (CWIS) at existing power plant facilities. Our management objectives, assessment endpoints, conceptual model, and generic analysis plan apply to all existing facilities. However, adapting the problem formulation process for a specific facility requires consideration of the permitting agency’s guidelines and level of regulatory concern, as well as site-specific ecological and technical differences. The facility-specific problem formulation phase is designed around the hierarchy of biolo gical levels of organization in the generic conceptual model and the sequence of cause-effect events and risk hypotheses represented by this model. Problem formulation is designed to be flexible in that it can be tailored for facilities where §316(b) regulatory concern is low or high. For some facilities, we anticipate that the assessment can be completed based on consideration of susceptibility alone. At the other extreme, a high level of regulatory concern combined with the availability of extensive information and consideration of costly CWIS mitigation options may result in the ecological risk assessment relying on analyses at all levels. Decisions on whether to extend the ecological risk assessment to additional levels should be based on whether regulatory or generator concerns merit additional analyses and whether available information is adequate to support such analyses. In making these decisions, the functional dependence between levels of analysis must be considered in making the transition to the analysis phase and risk estimation component of the ecological risk assessment. Regardless of how the generic analysis plan is modified to develop a facility-specific analysis plan, the resulting plan should be viewed as a tool for comparing representative species and alternative CWIS options by focusing on relative changes (i.e., proportional or percent changes) in various measures. The analysis plan is specifically designed to encourage consideration of multiple lines of evidence and to characterize uncertainties in each line of evidence. Multiple lines of evidence from different levels of analysis, obtained using both prospective and retrospective techniques, provide a broader perspective on the magnitude of potential effects and associated uncertainties and risks. The implications of the EPA’s recent (April 2002) proposed regulations for existing facilities on the applicability of this blueprint are briefly considered.

## INTRODUCTION

Impingement and entrainment at cooling-water intake systems (CWIS) are two sources of potential mortality for fish. Impingement occurs when fish are trapped or pinned by the force of the intake flow against the intake screens at the entrance of a facility's CWIS. Mortality can be high, but numerous technologies have been developed to successfully reduce at a reasonable cost both number of fish impinged and mortality of those fish that are impinged[1]. Entrainment occurs when fish eggs and larvae are taken into a facility's CWIS, pass through its heat exchanger, and are pumped back to the water body with the discharge from the facility. Mortality can approach 100% for sensitive species and life stages. However, for many species, mortality for those eggs and larvae entrained can be reduced when facilities are operated to reduce exposure of entrained organisms to potentially lethal high temperature, to large changes in temperature, and to toxic chemicals[2,3]. Substantially reducing the number of eggs and larvae entrained, however, is difficult to achieve at a reasonable cost for existing facilities with once-through cooling systems. This cost difference between mitigation technologies for entrainment as compared to impingement, in combination with the uncertain ecological impact created by entrainment, has led to a good deal of the difficulty and controversy surrounding §316(b) determinations.

The entire §316(b) text from the 1972 Clean Water Act is brief[4]: “Any standard established pursuant to Section 301 or Section 306 of this Act and applicable to a point source shall require that the location, design, construction, and capacity of cooling-water intake structures reflect the best technology available for minimizing adverse environmental impact.” The terms “best technology available” (BTA), “minimizing”, and “adverse environmental impact” (AEI) are not defined.

The U.S. Environmental Protection Agency (EPA) published §316(b) assessment guidelines in 1977 that were remanded in court due to procedural issues. Nonetheless, state regulators essentially followed the unofficial guidelines into the 1990s, with several hundred §316(b) determinations made during the 1970s and 1980s. In the absence of EPA regulations clearly defining AEI, BTA, or an assessment process, state and federal permitting authorities generated their own definitions on a case-by-case basis, relying on past decisions, administrative findings, scientific advances, and site-specific considerations. Several recent papers trace the history of §316(b) assessments[2,5,6,7]. Renewed interest in §316(b) assessments has been triggered by a 1995 Consent Decree that establishes a timetable for the EPA to propose and take final action with respect to addressing impacts from existing and new facilities. Final §316(b) regulations for CWIS for new facilities and proposed §316(b) regulations for large existing facilities have recently been released[4,8]. Our paper applies primarily to these large existing facilities.

In this paper, we briefly describe the EPA’s ecological risk assessment framework and recommend its use to more effectively guide §316(b) determinations. We focus on developing a blueprint for the problem formulation phase of the ecological risk assessments. This blueprint includes generic assessment endpoints, a conceptual model and analysis plan, and guidance on how to modify these three generic products to develop a facility-specific problem formulation plan. In addition, we discuss the transition from problem formulation to the analysis phase and risk estimation step of a §316(b) ecological risk assessment, methods of analysis available for §316(b) ecological risk assessments, and the implications of the EPA’s recent proposed regulations for existing facilities[8] on the applicability of this blueprint.

## THE EPA'S ECOLOGICAL RISK ASSESSMENT FRAMEWORK

The contribution of science to the §316(b) decision making could be increased if the §316(b) determination process adhered to an accepted overall risk assessment framework. All attempts to develop regulatory tools for §316(b) need to be viewed in the context of a dichotomy of definitions of AEI. Mayhew et al.[9] effectively summarize the history of eight definitions. This dichotomy has its basis, however, in a more fundamental difference than definitions of AEI. Differences in management objectives, assessment endpoints, and measures (defined below) for assessing CWIS effects cloud every step of the §316(b) regulatory effort[10,11].

The EPA ecological risk assessment process provides an effective framework for addressing these differences (Fig. 1)[12,13]. The EPA's Guidelines call for ecological risk assessments to be conducted in three sequential phases: problem formulation, analysis, and risk characterization. Alternative frameworks are used in other countries and by other organizations within the U.S.[14,15]. We have focused on the EPA framework because the EPA has responsibility for §316(b). In addition, others have recently suggested using the EPA ecological risk assessment framework for §316(b) assessments and for environmental decision making in general[2,16,17,18,19].

The EPA framework includes a hierarchy of terms, which we have adhered to throughout the paper[12,13].

**Management Goal.** A management goal is a general statement of the desired condition or direction of preference for the entity to be protected. It is often developed independently of any specific risk assessment, such as part of federal or state legislation. The enabling legislation for §316(b) is the Clean Water Act (1972). The management goal for this legislation [and thus for §316(b)] is "to protect and restore the chemical, physical, and biological integrity of the nation's waters."

**Management Objective.** A management objective is a specific statement about something one desires to achieve that includes an ecological entity targeted for protection, a direction of preference, and a decision context of place and time. It is commonly derived from a management goal and is focused on a particular regulation in the legislation. For the purpose of this paper, we define the ecological management objective relating to CWIS under §316(b) as follows: to maintain and ensure the sustainability of populations of species in the source water body and the beneficial uses these populations support[20,21,22].

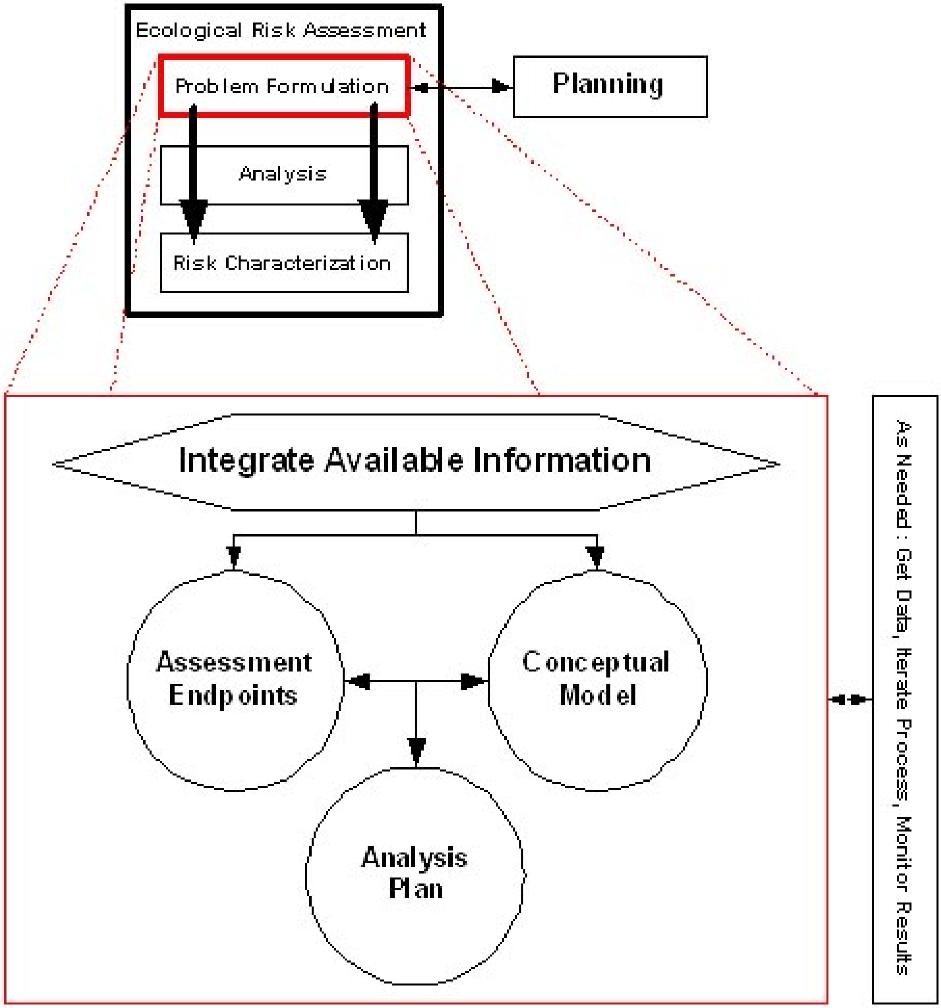

**FIGURE 1.** Flowchart illustrating the EPA's Ecological Risk Assessment process, including the three phases of problem formulation, analysis, and risk characterization, leading to communication of results and risk management[12].

An ecological management objective of maintaining sustainability of fish populations subjected to harvesting is favored by many scientists and used by most resource management agencies[23,24]. The focus on sustainability is favored for several reasons. First, this focus is premised on a view that the population level is the proper ecological level of biological organization for managing fishery resources. The reason for this is that all individual organisms have finite life spans; only populations and higher levels persist through time. As long as fish populations of concern are relatively stable and the mix of species present remains relatively constant, sustainability can be maintained in spite of the deaths of individuals. Second, while acknowledging uncertainty, fisheries resource management agencies believe they have the ecological understanding, experience, and scientific and sociopolitical tools to monitor, forecast, and adjust regulations sufficiently well to protect fish populations.

There are ample precedents in legislation and management guidelines for this focus on populations and even higher levels. The EPA's Guidelines for Ecological Risk Assessment[12] identifies "ecological relevance" as a key criterion for selecting management objectives, assessment endpoints, and specific entities. Regardless of how management objectives are established, those that explicitly define ecological values to be protected provide the best foundation for identifying actions to reduce risk and generating risk assessment objectives[25].

The focus on populations is also fundamental to natural resource management. The Magnuson-Stevens Fishery Conservation Act, for example, focuses on maintenance of sustainable yields from exploited populations. In fact, the concept of sustainable development implicitly focuses on populations and communities, because only populations and communities are persistent and therefore sustainable. Even for the Endangered Species Act, the management objective is preservation, conservation, and protection of endangered species, and not individual organisms.

**Assessment Endpoint.** An assessment endpoint is an explicit expression of what is to be protected. It is defined by an ecological entity and the entity's attributes, ideally including spatial and temporal extent. We define a hierarchy of population and community level assessment endpoints relating to CWIS under §316(b) later in this paper (Table 1).

**TABLE 1**
**Generic §316(b) Ecological Assessment Endpoints: Entities and Their Attributes**

| Level Within the Hierarchy of Management Objectives | Ecological Entity | Attributes of the Entity |
|---|---|---|
| Level 1 – Indigenous community | Fish and macroinvertebrate communities | Species composition; species richness; species diversity |
| Level 2 – Populations | All individual populations in the community | Population abundance; population reproductive success |
| Level 3 – Populations of species selected as representative species | Populations of representative species selected on a site-specific basis | Population abundance; population reproductive success |

**Measures.** EPA defines three classes of measures. Collectively, these measures are used to describe an assessment endpoint or factors affecting risk to that endpoint. *Measures of exposure* describe the existence and movement of a stressor in the environment and its contact or co-occurrence with the assessment endpoint or its surrogate. *Measures of effect* describe a change in an attribute of an assessment endpoint, or its surrogate, in response to a stressor to which it is exposed. *Measures of ecosystem characteristics and receptor characteristics* describe factors that influence the behavior and location of ecological entities and the distribution of a stressor, life-history characteristics of the assessment endpoint that may affect exposure to, or effect of, the stressor. For the purpose of this paper, we define measures relevant for §316(b) in terms of characteristics of the facility/CWIS, characteristics of the source water body, and characteristics of the fish inhabiting the water body.

**Risk Thresholds.** A risk threshold (or decision criterion, target, or benchmark) is defined as the level or value for a measure beyond which is thought to result in an unacceptable level of ecological risk. Risk thresholds can be useful at low levels of regulatory concern when used as part of a tiered screening process[7,12]. Examples are risk thresholds for measures of exposure, sensitivity, number killed by entrainment and impingement, and equivalent losses. Risk thresholds for measures at higher levels of ecological organization (i.e., at the population and community levels) will always be controversial and thus not useful for screening.

## THE EPA'S PROBLEM FORMULATION PHASE

Problem formulation, the first major phase of the EPA ecological risk assessment framework, is an extension of the planning process (Fig. 1). Planning and problem formulation provide the foundation for the following analysis and risk characterization phases of the ecological risk assessment. Whereas planning defines the overall responsibilities, available resources, and objectives for the ecological risk assessment, problem formulation identifies the cause-effect relationships, assessment endpoints, and measures that will be used in conducting the assessment.

Problem formulation results in three products[12]:

- Assessment endpoints that adequately reflect management goals, management objectives, and the ecosystem they represent;
- Conceptual model(s) that describe key relationships between stressors and assessment endpoints; and
- An analysis plan that documents the assessment endpoints, measures, and methods to be used in the analysis phase of the risk assessment.

The first step toward developing these products is to integrate available information. In practice, information needs are identified as part of the process of developing the above products, such that needed information is acquired and reviewed iteratively throughout the problem formulation phase. Each of the three products contains uncertainty. The explicit treatment of uncertainty during problem formulation is particularly important because it will have repercussions throughout the remainder of the ecological risk assessment.

The products of problem formulation are the scientific bases for analyzing exposure to, and effects of, a stressor on an ecological entity. Ensuring that these products are linked to the management objectives hierarchy is of utmost importance, so that the risk assessment yields indicators of risk relevant to the established values of concern.

## PROBLEM FORMULATION FOR §316(b) ECOLOGICAL RISK ASSESSMENTS

We describe problem formulation for ecological risk assessments of CWIS in two steps. First, we develop generic versions of the three products listed above that are appropriate for all §316(b) ecological risk assessments/determinations. Second, we describe a facility-specific process for problem formulation based on these three generic products.

### Generic Products for Problem Formulation in §316(b) Ecological Risk Assessments

On the surface, all §316(b) determinations might appear to be relatively straightforward. The source (CWIS), stressors (entrainment and impingement), receptors (typically fish and macroinvertebrates), and immediate effect (mortality) are well defined, have been studied in detail for decades, and are conceptually the same at all power plant facilities. As discussed earlier in this paper, however, the past quarter century history of §316(b) determinations amply demonstrates that environmental decision making that might appear to be relatively straightforward has commonly been controversial, time-consuming, expensive, and site specific. Value-based differences among regulators, generators, and other interested parties and site-specific differences at existing facilities explain why §316(b) determinations have not been straightforward. These differences also highlight why using the EPA's ecological risk assessment framework for §316(b) determinations merits consideration.

Final §316(b) regulations and guidelines for existing facilities need to be applicable nationwide and by water body type, as well as allowing for important facility-specific differences. Fortunately, the process of assessing entrainment and impingement impacts is fundamentally the same for all existing facilities. Consequently, generic versions of the products of problem formulation are needed that are appropriate as starting points to facilitate development of problem formulation plans for specific existing facilities.

## Generic Assessment Endpoints for §316(b)

Assessment endpoints for §316(b) determinations should be consistent with available guidance for selecting such endpoints, as discussed above, and with ecological principles and practice. Assessment endpoints that directly support the management objectives in the hierarchy may be established at both the community and population levels (Table 1). Assessment endpoints at the community level are more closely linked to the management goal. However, population-level endpoints are more directly linked to potential cause-effect consequences of entrainment and impingement losses [see next section on conceptual model for §316(b)]. Selecting endpoints at both these levels of biological organization is encouraged as part of a multiple-lines-of-evidence approach to reduce overall uncertainty in the risk assessment. Both retrospective and prospective methods of analysis are readily available at the population level, whereas only retrospective methods of analysis have been effective at the community level[26,27]. Nonetheless, community-level assessments alone may, in some cases, provide sufficient information for decision making, especially where extensive water body data are available and the level of regulatory concern is low.

Numerous field and laboratory studies and assessments of power plant impacts conducted on freshwater, estuarine, and marine systems over more than 3 decades have indicated that fish, and to a lesser extent nektonic macroinvertebrates, are the biological communities primarily susceptible to entrainment and impingement. Most other community components of a water body have either low exposure to the CWIS (e.g., benthic infauna and epifauna, vascular aquatic plants), or low sensitivity to effects from exposure (e.g., phytoplankton, zooplankton).

Recommended entities and their attributes for generic §316(b) assessment endpoints are listed in Table 1. These endpoints can be used to address the upper and lower levels of the management objectives hierarchy. The recommended community-level assessment endpoint is ecologically relevant by definition, because the endpoint is the community structure itself. Susceptibility to entrainment and impingement stresses at the community level is assured if, and only if, community attribute information used in the assessment is from, or relevant to, the water body segment affected by the CWIS. In contrast, ecological relevance and susceptibility to the CWIS is established for the population-level assessment endpoint by the process of selecting representative species[28] for the specific site in question.

Attributes of an assessment endpoint determine what to measure. Where direct measures of effect can be collected on the attribute(s) of concern (e.g., direct measures of population abundance), the assessment endpoint and measure of effect are the same[12]. Otherwise, surrogate measures of effect that are readily monitored or modeled[29] must be used (e.g., organism losses from entrainment and impingement), and the effect on the endpoint (i.e., population) must be projected, introducing further uncertainty into the risk assessment. Endpoints and associated measures, if carefully selected and defined, can provide a basis for comparing the effects of a range of stressors, with effects expressed in the same units[30]. For example, using susceptible representative species populations as assessment endpoints, rather than the water body community, has the additional advantage that measures of effect can be directly compared for various CWIS hardware or operational alternatives. Surrogate measures of population-level effects, such as entrainment and impingement losses, are useful for such relative comparisons, which do not require interpretation of effect on the assessment endpoint itself.

## *Generic Conceptual Model for §316(b)*

Factors specific to each facility and water body will influence the formulation of conceptual models appropriate for each specific §316(b) permitting action. However, commonality in the nature of the stressor and potential effects on assessment endpoints allow one to formulate a generic conceptual model for §316(b), which can be used to facilitate the development of facility-specific conceptual models and analysis plans. The generic conceptual model diagram (Fig. 2) shows the relationships among source, stressors, receptors and receptor responses, and processes influencing receptor responses. The figure also identifies risk hypotheses along the cause-effect path from stressors to potential responses by assessment endpoints.

Components of the generic conceptual model that should be described by the risk assessment are summarized below.

### Source

The CWIS is the source of the stressors addressed in §316(b) assessments. CWIS characteristics that affect the nature and magnitude of entrainment and impingement exposure include cooling-water flow, intake approach velocity, intake screen system design and location, and condenser temperature elevation ($\Delta T$). The CWIS hardware and operation, as well as the electric generating levels of the facility, may all influence entrainment and impingement exposure.

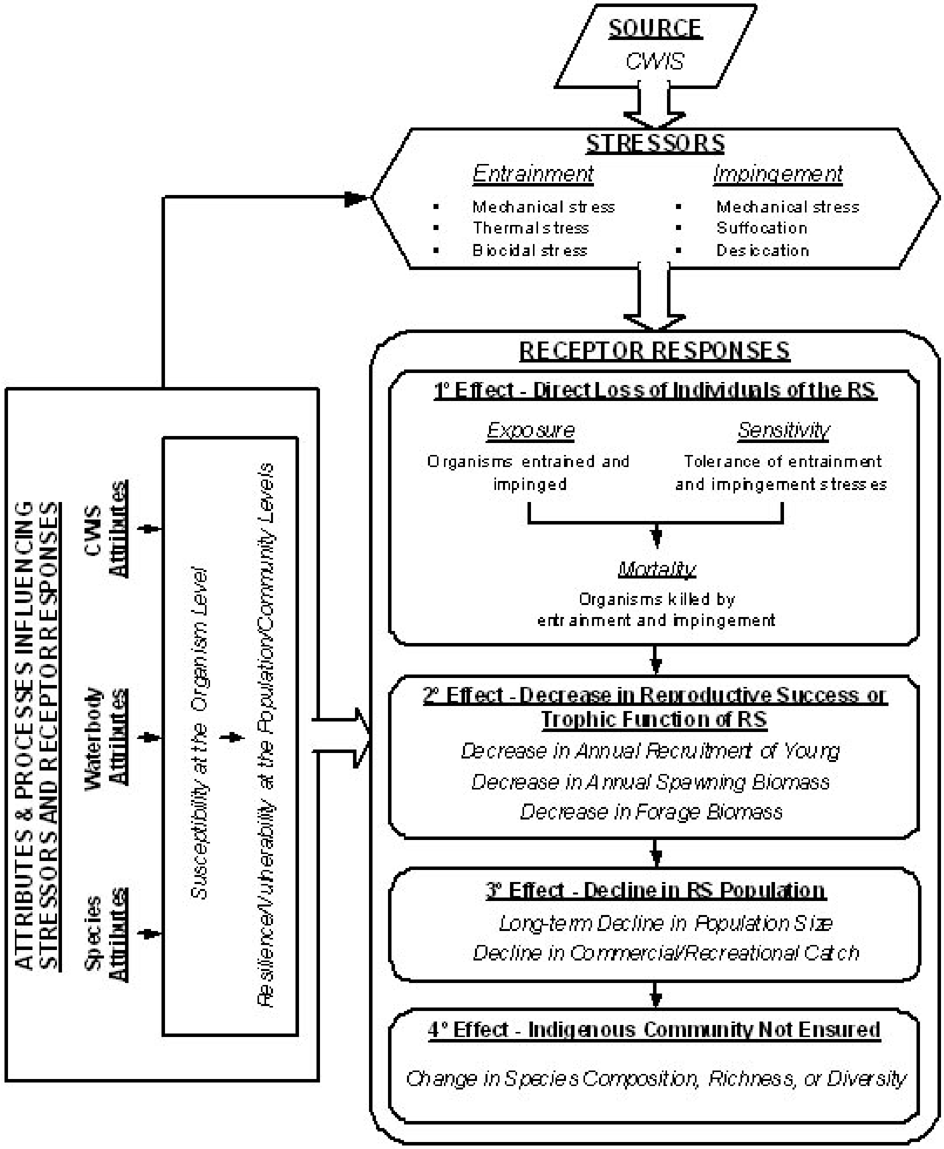

**FIGURE 2.** Generic conceptual model for §316(b) ecological risk assessments.

## *Stressors*

Entrainment and impingement are the two major categories of stressors that need to be considered in §316(b) risk assessments. Stresses from entrainment can be of three types: mechanical (e.g., pressure, shear forces), thermal (heat shock from condenser ΔT), and chemical (e.g., biocides such as chlorine). The process of impingement can expose organisms to mechanical (e.g., abrasion), suffocation, and desiccation stresses. Thermal stresses are influenced not only by CWIS design and operation, but also by seasonally varying ambient temperature characteristics of the water body.

### Receptors and Receptor Responses

The receptors for the §316(b) assessment are the populations of aquatic organisms that reside in the source water body, as represented by the representative species. Receptor response is primarily a function of factors that determine susceptibility[31] of the representative species by influencing either exposure or sensitivity, or both. The cause-effect linkages between stressors, receptors, and assessment endpoints is represented in Fig. 2 as a sequence of responses that constitute the hierarchy of risk hypotheses for the generic conceptual model. That is, the deaths of individuals of the representative species from entrainment and impingement (first-order effect) may cause a decline in reproductive success of their populations (second-order effect) that, in turn, could lead to long-term declines in population abundance (third-order effect) and changes in species composition at the community level (fourth-order effect). Measures of effect at any of these four levels may be used to characterize ecological risks.

### Processes Influencing Receptor Responses

Characteristics of the CWIS, water body (ecosystem), and representative species ultimately determine the effect of the CWIS on assessment endpoints by their influence on the nature and magnitude of entrainment and impingement stressors, exposure and sensitivity of the organisms to entrainment and impingement stresses, and vulnerability of the representative species to the entrainment and impingement losses incurred (Fig. 2).

## Generic Analysis Plan for §316(b)

The generic analysis plan for §316(b) is based on the §316(b) ecological management objectives hierarchy (Table 1), generic assessment endpoints, entities and their attributes (Table 1), and the generic conceptual model (Fig. 2). The plan is flexible and can be tailored for facilities for which the level of §316(b) regulatory concern is low or high. It is designed around the following six levels of analysis and associated scientific/management decision points (SMDP)[32]. The first five levels apply (potentially) to each representative species.

1. Describe exposure and susceptibility to entrainment and impingement by life stage,
2. Describe number killed annually by entrainment and impingement by life stage,
3. Describe annual equivalent losses,
4. Describe effects on annual reproductive success,
5. Describe multiyear effects on population abundance and beneficial uses, and
6. Describe multiyear effects on community composition.

These levels of analysis may be considered sequentially as indicated in the conceptual model (Fig. 2). However, one or more levels may be bypassed because of inadequate information or other reasons. An example is population projection modeling in the absence of retrospective estimates of effects on annual reproductive success. In either case, a scientific/management decision should be made prior to undertaking an analysis at a new level. This decision should be guided by consideration of regulatory guidelines, level of regulatory concern, and available information. In other words, why is analysis at this level needed, what will it contribute to the overall assessment, and can it be done in a scientifically credible manner with reasonable effort?

Regardless of how this generic plan is modified during the facility-specific §316(b) problem formulation process, the resulting analysis plan should be viewed as a blueprint for comparing alternative CWIS options and comparing representative species by focusing on relative changes (i.e., proportional or percent changes) in various measures. The analysis plan is specifically designed to encourage the regulatory agency to consider multiple lines of evidence in making a §316(b) determination.

## Facility-Specific §316(b) Problem Formulation

Figure 3 is a flowchart for problem formulation for existing facilities. Adapting the generic products to develop a facility-specific analysis plan requires consideration of specific regulations and guidelines of the permitting agency, as well as site-specific ecological and technical differences including:

- Characteristics of the facility and its CWIS,
- Characteristics of the water body from which the CWIS withdraws water,
- Characteristics of the fish species in the water body,
- Magnitude of entrainment and impingement losses, and
- Quantity and quality of information available to characterize the preceding four items and to evaluate the ecological consequences of entrainment and impingement losses.

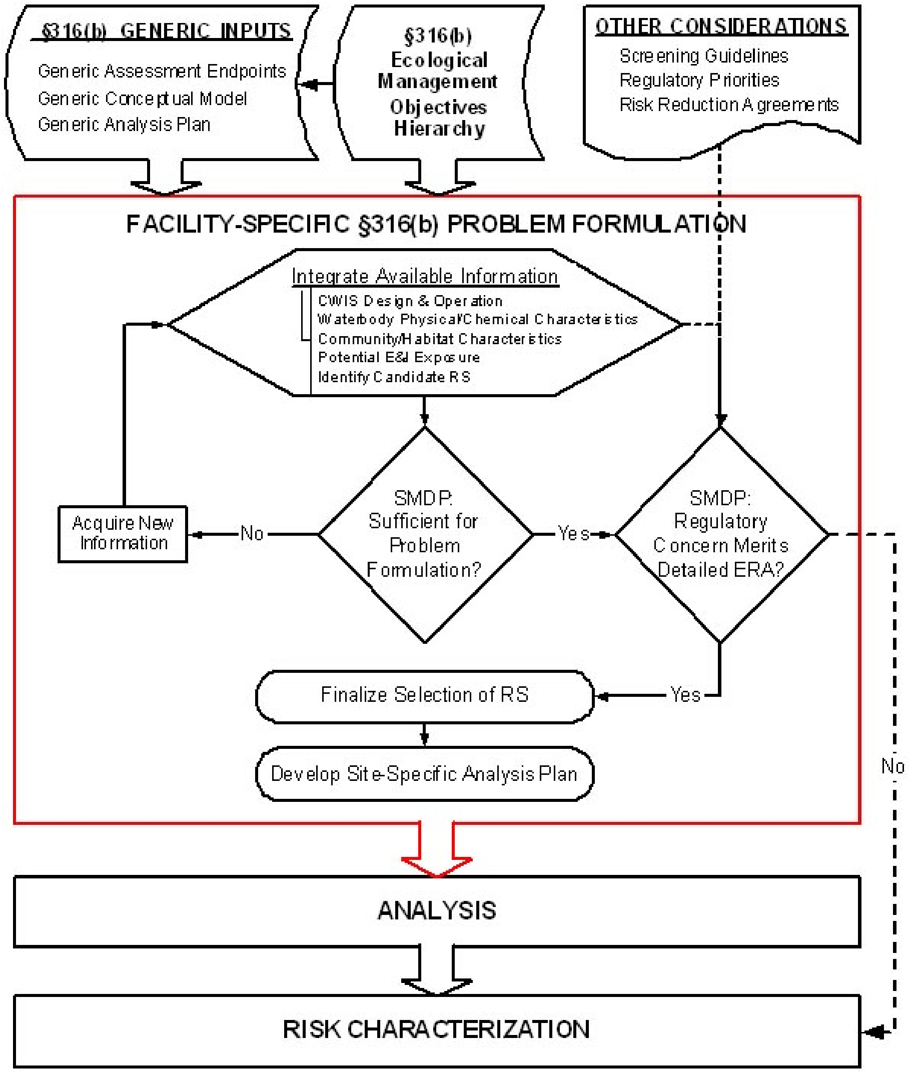

**FIGURE 3.** Flowchart of the facility-specific problem formulation process for §316(b) ecological risk assessments.

The facility-specific §316(b) problem formulation process (Fig. 3) is based on the ecological management objectives hierarchy and the generic assessment endpoints, conceptual model, and analysis plan described in the preceding sections, and includes two SMDP. The facility-specific problem formulation process is intended to be iterative, involving both planning and cycling through the following five steps as needed.

- Integrate available information;
- Determine if available information is sufficient to support problem formulation, including selection of assessment endpoints, representative species, and preparation of a site-specific analysis plan;
- Determine if the level of regulatory concern for entrainment and impingement losses merits a detailed ecological risk assessment;
- Select representative species to be the focus of the risk assessment; and
- Complete and document the facility-specific analysis plan.

### Integrate Available Information

During the problem formulation process (Fig. 3), site-specific information would be compiled and evaluated to develop an analysis plan that addresses some or all of the risk hypotheses identified in the generic conceptual model. The process would typically be iterative, in which information is integrated with evolving plans for analysis, new information requirements are identified, and the process of compiling, evaluating, and integrating that information is repeated. Experienced risk assessors recognize the importance of performing this task well: "The foundation for problem formulation is based on how well available information on stressor sources and characteristics, exposure opportunities, characteristics of the ecosystem potentially at risk, and ecological effects are integrated and used"[12].

At this point in the risk assessment, the focus of compilation and review is on information needed for preliminary evaluation of the level of regulatory concern, selection of the representative species to serve as assessment endpoints, and preparation of a site-specific analysis plan. A second, more focused, compilation and evaluation of available information occurs at the beginning of the analysis phase to support tasks in analysis and risk characterization (Fig. 1). Information necessary at this point, however, is limited to a combination of CWIS design and operating data, as well as information on the source water body and its aquatic inhabitants. Examples of specific information needed for this step are:

- Design and construction of the CWIS, including dimensions, capacities, and equipment for reducing entrainment and impingement losses;
- Typical operation of the CWIS, including seasonal patterns in cooling-water flow, operation of the intake screens, and operation of the equipment for reducing entrainment and impingement losses;
- Physical characteristics of the source water body, including size, depths, and general hydrologic conditions;
- Environmental conditions within the source water body, including physical, chemical, and habitat characteristics; and

- Composition and status of the aquatic community expected to be in the vicinity of the intake, with special focus on those components typically most susceptible to entrainment and impingement (e.g., fish and macroinvertebrates).

### Determine if Available Information is Sufficient to Support Site-Specific Problem Formulation

Following compilation and review of available information, the §316(b) problem formulation process (Fig. 3) includes a determination as to whether the currently available information is sufficient to continue with the problem formulation phase. Typically, available information necessary to complete this step will be obtained from the plant operator, from the natural resource management agency responsible for the source water body, and from the general literature. If available data are deemed insufficient (e.g., no information on fish species occurring in the vicinity of the intake), a work plan should be developed and implemented to gather such information.

### Determine if Level of Regulatory Concern Merits a Detailed Ecological Risk Assessment

When available information is deemed sufficient to support problem formulation, the regulatory agency determines if the level of regulatory concern for entrainment and impingement losses merits a detailed ecological risk assessment. More than 500 existing electric generating stations with CWIS are subject to regulation under §316(b)[8]. However, based on the results of detailed §316(b) assessments conducted at several larger generating stations throughout the country, it is likely that many of these existing facilities have little risk of AEI to aquatic populations or the community in the source water body. Therefore, an important function of using the ecological risk assessment process for §316(b) determinations is to provide a screening mechanism that identifies those facilities that pose little risk of AEI. Such low-risk facilities, then, will not be required to conduct a detailed analysis of potential CWIS effects, but rather the ecological risk assessment process will proceed directly to a limited risk characterization (Fig. 3). A brief summary report will then document the conclusions of the assessment and include the basis for the conclusions that will be part of the regulatory record.

The bases for screening would include the preliminary indications of potential risks obtained by integrating available information, as well as other considerations brought by parties to the permitting process. Such considerations may include screening guidelines codified in regulations, priorities established in written regulatory policies, CWIS fish protection technologies existing at the facility, and agreements for risk reduction actions that have been offered by the permit applicant (Fig. 3). It seems reasonable to expect that the ecological risk assessment for those facilities that screen out at this point in the process could be completed in a few months with relatively small commitment of agency time and resources. Inclusion of such a screening process will help to ensure that the limited resources of the regulatory agencies are most effectively utilized to address §316(b). It is only when the regulatory agency deems the risk of AEI sufficiently high that a commitment of agency and utility resources is warranted to complete a full ecological risk assessment (Fig. 1).

It is up to the discretion of the regulatory agency, acting in consultation with all stakeholders, to determine whether a detailed ecological risk assessment is warranted for any specific facility. Some site-specific factors that might be considered in such a determination include:

- Capacity of the intake relative to the magnitude of the source water body,
- Frequency of operation of the CWIS (e.g., rarely used),
- Incorporation of CWIS features that minimize the use of cooling water (e.g., cooling towers),
- Incorporation of CWIS features that minimize exposure of aquatic organisms (e.g., barriers or screening),
- Incorporation of CWIS features that maximize survival of exposed aquatic organisms (e.g., fish return systems),
- Water quality in the vicinity of the intake that minimizes aquatic organism exposure (e.g., anoxic area), and
- Health of the communities of potentially exposed aquatic organisms in the vicinity of the intake provides no evidence for concern (e.g., biocriteria).

Table 2 provides an expanded list of selected characteristics of the facility/CWIS, the water body, and representative species that may be considered in the screening process, as well as in determining the level of assessment complexity. These characteristics influence available information and level of regulatory concern and would provide a basis for a tiered approach to risk assessment.

## *Select Representative Species*

The representative species are the ecological entities that are the focus of the §316(b) ecological risk assessment. Changes in selected attributes of these species become the measures of effect that are at the heart of the assessment.

**TABLE 2**
**Attributes to be Considered in a Tiered Approach for Determining the Appropriate Assessment Levels for §316(b) Ecological Risk Assessments for Existing Facilities**

| Assessment Detail/Level | Low | | High |
|---|---|---|---|
| **Facility/CWIS Attributes** | | | |
| Intake flow per day | Low | | High |
| Proportional intake flow (%) | Low | | High |
| Intake velocity | Low | | High |
| Years of operation remaining | Few | | Many |
| Attributes that minimize exposure or maximize survival | Many | | Few |
| **Water Body Attributes** | | | |
| Critical habitat function | Low | | High |
| Size | Large | | Small |
| Other stressors[1] | Low | | High |
| Value of beneficial uses[2] | Low | | High |
| Apparent community health | High | | Low |
| **Fish Attributes** | | | |
| Life history strategy[3] | E | | O, P |
| Number of species susceptible | Few | | Many |
| **Obtainable Information** | | | |
| Quality and quantity | Low | | High |
| Levels of biological organization[4] | Ind | Ind and YC | Ind, YC, and Pop |
| **Regulatory Priority** | Low | | High |

[1]Index of strength of other anthropogenic and nonanthropogenic stresses on the water body, e.g., fishing, water quality, temperature regime, drought.
[2]Economic, social, and cultural value of commercial, recreational, and subsistence fisheries and other beneficial uses combined.
[3]Key to life-history strategies: E = equilibrium (e.g., largemouth bass); O = opportunistic (e.g., bay anchovy, threadfin shad); P = periodic (e.g., striped bass) [Winemiller, K.O. and Rose, K.A. (1992) *Can. J. Fish. Aquat. Sci*. **49**, 2196–2218.]
[4]Key to levels of biological organization: Ind = individual; YC = year class; Pop = population.

Fish species susceptible to entrainment and impingement losses may or may not be identified in the current NPDES/ SPDES permit for an existing facility. If they are not, regulators and the generator need to address this issue. Guidelines and assumptions for selection of representative species are available[27,33]. Typically, representative species include those that are susceptible to entrainment and impingement, are representative of important functional roles in the community, are representative of important beneficial uses (e.g., commercial and recreational fisheries), or are species of special concern. The intent of focusing on the representative species is to ensure that potential consequences of entrainment and impingement losses to the aquatic community of the source water body as a whole are addressed.

## *Complete and Document Facility-Specific Analysis Plan*

Following final selection of the representative species, the facility-specific analysis plan is completed and documented (Fig. 3). In addition to the basic requirements of any analysis plan, as discussed above, the following two topics are of particular importance for the facility-specific analysis plan for §316(b) assessments.

- For each representative species, consider the levels of analysis that available information will support and that regulatory concern merits. Because analysis at each additional level will require additional resources (money, time, technical expertise), both available information for the species and level of regulatory concern for the species should be evaluated.
- Select CWIS options of interest to regulators, generators, or interested parties. Selection can be based on considering the extent to which each option is likely to reduce entrainment and impingement losses based on experience at other facilities, as well as economic and other impacts.

The facility-specific analysis plan summarizes the problem formulation phase of the §316(b) ecological risk assessment. It is the blueprint for how the analysis for that existing facility relates to the management objectives and to the NPDES/SPDES permit decisions that must be made. The facility-specific analysis plan needs to be documented for the public record in the final ecological risk assessment report to regulators.

We deliberately have not proposed risk thresholds for measures at any level of ecological organization. Proposing such values would have shifted the focus of this paper from proposing a blueprint for the problem formulation phase of §316(b) ecological risk assessments to justifying the thresholds we selected. Selection of these thresholds is only partly a scientific process. In making a §316(b) determination for an existing facility, agency regulators are ultimately the ones who operationally define AEI for that facility (Fig. 1). Scientists must be willing and able, however, to make species- and facility-specific estimates of uncertainty and risk of adverse effects, and then describe and communicate these risks to agency risk managers. Science alone is not and never will be in a position to provide an acceptable definition for AEI under §316(b) or for any other type of ecosystem modification[19,34,35,36]. Final determination of AEI under §316(b) is made by the regulatory agency based on joint consideration of risks of AEI and other factors, as strongly emphasized elsewhere in this volume[16,37,38,39,40,41].

## TRANSITION FROM PROBLEM FORMULATION TO ANALYSIS AND RISK ESTIMATION

The analysis phase follows the problem formulation phase in the EPA's ecological risk assessment framework (Fig. 1). The two products of the analysis phase are an exposure profile and a stress-response profile[12]. The generic conceptual model for §316(b) developed in problem formulation represents a chain of cause-effect ecological connections (Fig. 2). The functional dependence between some of the levels in this model merits emphasis and should be reflected in making the transition from problem formulation to actually performing analyses. This organization also parallels the organization for prospective methods under the headings of individual loss, fractional loss, and population projections[26,27].

- Describe susceptibility to entrainment and impingement, where susceptibility includes exposure, sensitivity, and mortality due to entrainment and impingement;
- Describe annual number killed by entrainment and impingement, and the resulting effects on annual equivalent losses; and
- Describe effects on annual recruitment, and the resulting multi-year effects on population abundance and beneficial uses.

We recommend for §316(b) assessments that characterization of exposure, summarized in an exposure profile, be replaced by characterization of susceptibility, summarized in a susceptibility profile, which is a combination of the exposure and stressor-response profiles. Fig. 4 highlights the conceptual limitations in assuming that exposure and susceptibility are equivalent. We propose defining an index of susceptibility for each representative species as a weighted sum of species-specific attributes, facility/CWIS attributes, and water body attributes that are applicable for that species (Fig. 2), as part of a multiple-lines-of-evidence approach. As an example, the likelihood of fish being withdrawn by hydropower intakes was determined primarily by the ecological zone from which water is withdrawn by hydropower intakes (e.g., littoral, pelagic, bathy-pelagic, etc.) and secondarily by the life-history strategy of the affected fish species[42].

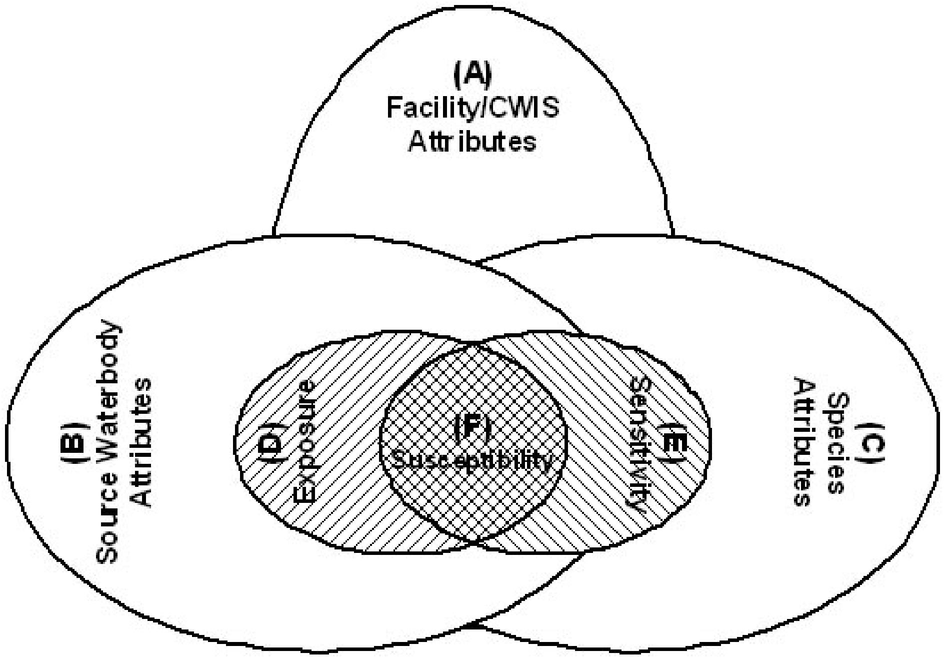

**FIGURE 4.** Venn diagram for conceptualizing characteristics that influence susceptibility to entrainment and impingement. The three ovals represent characteristics of (A) the facility/CWIS, (B) the source water body, and (C) the fish species in the water body. Oval (D) represents the reduced subset of characteristics from (A), (B), and (C) that together determine exposure. Oval (E) represents the reduced subset of characteristics from (A), (B), and (C) that together determine sensitivity. The intersection of ovals (D) and (E) [i.e., area (F)] represents mortality due to entrainment and impingement. Technological advances continue to modify CWIS characteristics at existing facilities, resulting in reductions in the size and overlap of ovals (D) and (E), and thus reductions in entrainment and impingement mortality and annual losses.

Describing the number killed annually by entrainment and impingement (by species and life stage) is an important step for proceeding to any of the higher levels of analysis. If the numbers killed annually with the existing CWIS are not of regulatory concern, the regulatory decision may be to summarize the results of analysis up to that point and go to risk estimation. If the numbers killed are of regulatory concern, it is essential from the ecological perspective that the analysis progress to the next higher level to describe the resulting annual equivalent losses by species. The numbers of eggs and larvae killed by entrainment, while they may be very large numbers, do not reflect the very low natural survival rates, which can differ substantially by life stage and species. Compared to survival rates with which the public is familiar, survival rates for fish eggs and larvae of species experiencing entrainment and impingement losses are extremely low[43,44]. Extrapolating entrainment and impingement losses of these early life stages to equivalent losses (as numbers or biomass) at some older life stage (e.g., juvenile age 0, age 1, or adults) avoids the problem of mixing “apples and oranges” and provides a measure of effect of greater relevance both ecologically and socially.

Describing effects on annual recruitment and on the resulting multiyear effects on population abundance and beneficial uses typically involves both retrospective and prospective methods for those existing facilities where analysis at these higher levels is merited. These extrapolations to a large extent are independent of extrapolating annual entrainment and impingement losses to annual equivalent losses. For both retrospective and prospective methods, describing multiyear effects at the population level, as compared to describing annual effects on reproductive success, will be more relevant ecologically and socially because they reflect cumulative effects of entrainment and impingement losses over a period of years and are closer to the assessment endpoint. On the other hand, measures of effect at the population level are unavoidably more uncertain than measures of effect at the year-class level (Table 2).

## Methods of Analysis Available for §316(b) Ecological Risk Assessments

Given the above discussion of levels of analysis, a brief overview of methods available to estimate measures at these various levels of analysis is constructive. Depending on the magnitude of perceived effects and the desires and capabilities of the regulatory agencies, generators, and interested parties, a variety of measures (and associated methods to estimate these measures) have been used to describe susceptibility and ecological effects at the individual, year-class, population, and community levels. The methods have developed and evolved over the past 30 years and can be broadly grouped into two categories, prospective and retrospective.

Prospective methods attempt to forecast what effects cooling-water withdrawals will have by combining information on the magnitude of actual or predicted entrainment and impingement losses with information on the life history characteristics and population dynamics for each susceptible species. This category of methods is consistent with the EPA’s category of process modeling techniques[12]. With prospective methods, magnitude of entrainment and impingement loss is typically based on site-specific information on the density of susceptible life stages in the vicinity of the intake and the mortality rates associated with the entrainment and impingement processes. Where necessary, information on the life history characteristics and population dynamics of a species can usually be obtained from the scientific literature for the same or for closely related species. The range of prospective methods extends from generally qualitative to highly sophisticated quantitative models of population dynamics. Generally, estimation of effects of cooling-water withdrawals using prospective methods is conducted in a phased manner beginning with the simplest methods. The more complex modeling exercises are typically limited to those cases where the potential for adverse population effects is expected to be high and, consequently, the concern of the regulatory agencies and the public at large is heightened.

Retrospective methods include those procedures that analyze empirical data from the source water body for evidence that entrainment and impingement losses may be having a demonstrable effect at the year-class and population levels. This category of methods is consistent with the EPA's category of empirical modeling techniques[12]. In general, retrospective methods provide direct measures of potential effects. These methods become increasingly applicable the greater the number of years an existing facility has been operating. It is important to remember that use of retrospective methods requires assessing to what extent observed effects may be caused by entrainment and impingement losses vs. unrelated natural changes in the ecosystem and other anthropogenic stresses.

## THE EPA'S PROPOSED REGULATIONS FOR CWIS AT EXISTING FACILITIES

The recently proposed regulations for existing facilities[8] shift the emphasis for 316(b) determinations from an assessment process that characterizes risk of AEI to an assessment process that characteristics benefits and costs of alternative technologies to meet technology-based performance standards for CWIS. The primary proposed assessment endpoints are the CWIS itself, percent reduction in impingement mortality, and percent reduction in number entrained. Regardless of the regulatory emphasis, the great majority of the blueprint for problem formulation proposed in this paper is applicable for 316(b) determinations at existing facilities. Two implications, however, are obvious. First, the focus of assessments will shift toward comparing CWIS options and representative species and away from estimating multiyear population effects for the existing CWIS. Second, methods of analysis will shift toward describing exposure, susceptibility, entrainment, and impingement losses by life stage and equivalent losses.

In conclusion, the problem formulation process for §316(b) ecological risk assessments we propose capitalizes on the considerable body of scientific knowledge and regulatory experience that has accumulated from §316(b) determinations over the past 3 decades. Our blueprint for existing facilities provides the structure and process that can substantially improve the current repermitting procedure for regulators, generators, and other interested parties.

For existing facilities, both prospective and retrospective methods should be used to provide multiple lines of evidence. Multiple lines of evidence from different levels of analysis, and using both prospective and retrospective methods, provide a broader perspective concerning magnitude of effects and associated uncertainties and risks.

## ACKNOWLEDGMENTS

The authors thank D.E. Bailey, J. Kadvany, J. Loos, and W.A. Richkus for their constructive comments during the preparation and peer review of this paper.

## REFERENCES AND ENDNOTES

1. Taft, E.P. (2000) Fish protection technologies: a status report. *Environ. Sci. Policy* **3(Suppl. 1),** S349–S360.
2. Dey, W.P., Jinks, S.M., and Lauer, G.J. (2000) The §316(b) assessment process: evolution towards a risk-based approach. *Environ. Sci. Policy* **3(Suppl. 1),** S15–S24.
3. Mayhew, D.A., Jensen, L.D., Hanson, D.F., and Muessig, P.H. (2000) A comparative review of entrainment survival studies at power plants in estuarine environments. *Environ. Sci. Policy* **3(Suppl. 1),** S295–S302.
4. USEPA (United States Environmental Protection Agency). (2001) National Pollutant Discharge Elimination System. Final Regulations Addressing Cooling Water Intake Structures for New Facilities; Final Rule. Federal Register, Environmental Documents, December 18, 2001, pp. 65255–65345. www.epa.gov/owm
5. Anderson, W. and Gotting, E. (2001) Taken in over intake structures? Section §316(b) of the Clean Water Act. *Columbia J. Environ. Law* **26**, 1–79.
6. May, J.R. and van Rossum, M.K. (1995) The quick and the dead: fish entrainment, entrapment, and the implementation and application of Section §316(b) of the Clean Water Act. *Vt. Law Rev*. **20(2),** 375–493.
7. Nagle, D.G. and Morgan, J.T., Jr. (2000) Forward. A draft regulatory framework for analyzing potential adverse environmental impact from cooling water intake structures. *Environ. Sci. Policy* **3(Suppl. 1),** ix–xiv.
8. USEPA (United States Environmental Protection Agency). (2002) National Pollutant Discharge Elimination System—Proposed Regulations to Establish Requirements for Cooling Water Intake Structures at Phase II Existing Facilities; Proposed Rule. Federal Register, Environmental Documents, April 9, 2002. pp. 17221–17225 and 17171–17220. www.epa.gov/owm .
9. Mayhew, D.A., Muessig, P.H., and Jensen, L.D. (2002) Adverse environmental impact (AEI): 30-year search for a definition. In Defining and Assessing Adverse Environmental Impact Symposium 2001. *TheScientificWorldJOURNAL* **2(S1)**, 21–29.
10. EPRI (Electric Power Research Institute). 2002. Evaluating the Effects of Power Plant Operations on Aquatic Communities. An Ecological Risk Assessment Framework for Clean Water Act §316(b) Determinations. EPRI, Palo Alto, CA. EPRI Report 1000758
11. Gordon, D.K. and Super, R.W. (2002) Minimizing adverse environmental impact: how murky the waters. *TheScientificWorldJOURNAL,* in press.
12. USEPA (United States Environmental Protection Agency). (1998) Guidelines for Ecological Risk Assessment. EPA/630/R-95/002F.

13. USEPA (United States Environmental Protection Agency). (2001) Planning for Ecological Risk Assessment: Developing Management Objectives. EPA/630/R-01/001A.
14. McCarty, L.S. and Power, M. (2000) Approaches to developing risk management objectives: an analysis of international strategies. *Environ. Sci. Policy* **3**, 299–304.
15. Power, M. and McCarty, L.S. (1998) A comparative analysis of environmental risk assessment management frameworks. *Environ. Sci. Technol*. **32**, 224A–231A.
16. Bailey, D. and Bulleit, K. (2002) Defining adverse environmental impact: a fisheries management approach. In Defining and Assessing Adverse Environmental Impact Symposium 2001. *TheScientificWorldJOURNAL* **2(S1)**, 147–168.
17. Gentile, J.H. and Harwell, M.A. (1998) The issue of significance in ecological risk assessments. *Human Ecol. Risk Assess*. **4(4),** 815–828.
18. Harwell, M. and Gentile, J. (2002) Overcoming barriers to the use of models in decision making. In *Using Models for Environmental Management*. Dale, V.H., Ed. Springer-Verlag, New York, in press.
19. Van Winkle, W., and Kadvany, J. Modeling fish entrainment and impingement impacts and the policy-science bridge. In *Using Models for Environmental Management*. Dale, V.H., Ed. Springer-Verlag, New York, in press.
20. The ecological management objective is expressed here using the population level because the population level is the lowest level of biological organization that persists through time, and because it is more directly linked than higher levels to potential consequences from impingement and entrainment losses. However, the broader management objective of protecting the communities, of which the populations are a part, is implicit in this definition.
21. "In a democratic society, the values represented in Federal law are often good indicators of widely held values. Except for endangered species, no case was found in which an individual nonhuman organism, or even a small number of individuals, was protected by a regulatory decision. However, effects somewhere between the individual and population levels, such as widespread mortality in fish or birds, have been used as the basis for decisions" (See endnote 24).
22. The controversy associated with some §316(b) determinations involves the difference between this ecological management objective and the alternative management objective of minimizing entrainment and impingement losses. This alternative objective reflects the societal value that it matters how we kill fish, not just that they are being killed. For some regulatory agencies and interested parties, the management objective is to minimize 'collateral losses' associated with entrainment and impingement at CWISs (see endnotes 4 and 8).
23. National Research Council. (1998) Improving Fish Stock Assessments. National Academy Press, Washington, D.C.
24. USEPA (United States Environmental Protection Agency). (1997) Priorities for Ecological Protection: An Initial List and Discussion Document for EPA. EPA/600/S-97/002.
25. McDaniels, T. (2002) Creating and using objectives for ecological risk management. *Environ. Sci. Policy* **3**, 299–304.
26. EPRI (Electric Power Research Institute). (1999) Catalog of Assessment Methods for Evaluating the Effects of Power Plant Operations on Aquatic Communities. EPRI, Palo Alto, CA, TR-112013.
27. EPRI (Electric Power Research Institute). (2002) Evaluating the Effects of Power Plant Operations on Aquatic Communities. Guidelines for Selection of Assessment Methods. EPRI, Palo Alto, CA. TR-1005176, in press.
28. Representative species (RS)—Species selected during problem formulation on a site-specific basis that are the focus of the ecological risk assessment. Equivalent terms used in other reports and published papers are focal species (FS), representative indicator species (RIS), representative and important species (RIS), species of concern (SOC).

29. However, while established measurement protocols are convenient and useful, they do not justify establishing assessment endpoints that are equivalent to the readily available measure. Data availability alone is not an adequate criterion for selection of assessment endpoints (see endnote 12).
30. Barnthouse, L.W., Suter, G.W., II, and Rosen, A.E. (1990) Risks of toxic contaminants to exploited fish populations: influence of life history, data uncertainty, and exploitation intensity. *Environ. Toxicol. Chem*. **9**, 297–311.
31. The reader should note that selection criteria for the representative species include susceptibility to the entrainment and/or impingement stressors, so that representative species would generally be more susceptible than the average for all the fish or macroinvertebrate populations comprising the community.
32. SMDP is USEPA'S term for Scientific/Management Decision Point, defined as: "A time during the ecological risk assessment when a risk assessor communicates results or plans at that stage to a risk manager. The risk manager decides if information is sufficient to proceed with risk management strategies or whether more information is needed to characterize risk" [USEPA (United States Environmental Protection Agency). (1999) Risk Assessment Guidance for Superfund: Volume 3 – (Part A, Process for Conducting Probabilistic Risk Assessment). Draft, Revision No. 5. U.S. Environmental Protection Agency, Washington, D.C. www.epa.gov/superfund/pubs.htm
33. USEPA (United States Environmental Protection Agency). (1977) Guidance for Evaluating Adverse Environmental Intake Structures on the Aquatic Environment: Section 316(b). (Draft). U.S. Environmental Protection Agency, Washington, D.C.
34. Lackey, R.T. (1994) Ecological risk assessment. *Fisheries* **19(9),** 14–18.
35. Lackey, R.T. (1998) Fisheries management: integrating societal preference, decision analysis, and ecological risk assessment. *Environ. Sci. Policy* **1**, 329–335.
36. Lackey, R.T. (1999) Salmon policy: science, society, restoration, and reality. *Environ. Sci. Policy* **2**, 369–379.
37. Kadvany, J. (2002) Decision theory and adverse environmental impacts in Section §316(b) of the Clean Water Act. In Defining and Assessing Adverse Environmental Impact Symposium 2001. *TheScientificWorldJOURNAL* **2(S1)**, 106–138.
38. McLean, R., Richkus, W.A., Schreiner, S.P., and Fluke, D. (2002) Maryland power plant cooling-water intake regulations and their application in evaluation of adverse environmental impact. In Defining and Assessing Adverse Environmental Impact Symposium 2001. *TheScientificWorldJOURNAL* **2**(**S1**), 1–11.
39. Strange, E.M., Lipton, J., Beltman, D., and Snyder, B. (2002) Scientific and societal considerations in selecting assessment endpoints for environmental decision-making. In Defining and Assessing Adverse Environmental Impact Symposium 2001. *TheScientificWorldJOURNAL* **2**(**S1**), 12–20.
40. Veil, J.A., Puder, M.G., Littleton, D.J., and Johnson, N. (2002) A holistic look at minimizing adverse environmental impact under Section §316(b) of the Clean Water Act. In Defining and Assessing Adverse Environmental Impact Symposium 2001. *TheScientificWorldJOURNAL* **2(S1)**, 41–57.
41. Wells, A.W. and Englert, T.L. (2002) AEI assessments: a consultant's perspective. In Defining and Assessing Adverse Environmental Impact Symposium 2001. *TheScientificWorldJOURNAL* **2(S1)**, 190–203.
42. Pavlov, D.S., Lupandin, A.I., and Kostin, V.V. (1999) Downstream migration of fish through dams of hydroelectric power plants. Moscow, Nauka Russian Academy of Sciences (in Russian; translation available from G.F. Cada or C.C. Coutant, Oak Ridge National Laboratory, Oak Ridge, TN 37831).
43. Houde, E.D. (1987. Fish early life dynamics and recruitment variability. *Am. Fish. Soc. Symp*. **2**, 17–29.
44. Miller, T.J., Crowder, L.B., Rice, J.A., and Marshall, E.A. (1988) Larval size and recruitment mechanisms in fishes: toward a conceptual framework. *Can. J. Fish. Aquat. Sci.* **45**, 1657–1670.

## BIOSKETCHES

**Webster Van Winkle** is self-employed with Van Winkle Environmental Consulting Co. He retired from Oak Ridge National Laboratory after 27 years in the Environmental Sciences Division. His research interests include data analysis and development and application of models as part of applied research projects and environment assessments involving aquatic ecosystems, fish populations in particular.

**William P. Dey** is

**Steven M. Jinks** is

**Mark S. Bevelhimer**, Ph.D., is a Research Scientist at Oak Ridge National Laboratory. He received his M.S. from Ohio State University and his Ph.D. from the University of Tennessee. He has 18 years experience in aquatic ecology/fisheries biology. Dr. Bevelhimer's work has been a combination of field observation, laboratory experimentation, and computer modeling. He has used laboratory and field studies to investigate the effects of environmental changes on fish growth, contaminant accumulation, food habits, movement, and population dynamics. He has supplemented his empirical research with simulation modeling to examine fish movement, growth, food consumption, and contaminant uptake. Modeling experience includes the development and application of bioenergetics models of fish growth, individual-based population models, and hydrologic models of stream flow and water quality. Most recently he has been using these skills to investigate the impacts of powerplant operations on resident and miagratory fish.

**Charles C. Coutant,** Ph. D., is Distinguished Research Scientist, Oak Ridge National Laboratory. Dr. Coutant has conducted laboratory and field research on the effects of power plant cooling systems on aquatic life since 1959 in the Delaware River (PA/NJ), Columbia River (WA/OR), Tennessee Valley reservoirs (TN), and has conducted power plant assessments for facilities in Oregon, Michigan, New York, New Jersey, Georgia, Sweden, and New Zealand. He has been an advisor on power plant effects to the International Atomic Energy Agency and UNESCO and has authored USEPA water temperature criteria. Dr. Coutant was past President of the American Fisheries Society. His current research interests lie in fish behavior as related to water intakes and fish bypasses for thermal electric power plants and hydropower dams, with continuing interest in water temperature effects on fish and temperature in the aquatic landscape. Awards and honors received include the Award of Excellence, Southern Division of the American Fisheries Society and Distinguished Service Award of the American Fisheries Society.
